# Current issues of tandem mass spectrum (MS2)-based glycoproteomics and efforts to complement them

**DOI:** 10.1016/j.bbadva.2025.100158

**Published:** 2025-03-14

**Authors:** Hiroyuki Kaji

**Affiliations:** Institute for Glyco-core Research (iGCORE), Nagoya University, Japan

**Keywords:** Glycoproteomics, Glycosylation-site mapping, Site-specific glycan composition, Liquid chromatography/mass spectrometry (LC/MS), Human Glycome Atlas Project (HGA)

## Abstract

With the development of liquid chromatography/mass spectrometers that support proteomics and the associated development of analytical methods and data analysis software, the number of proteins that can be identified and quantified in a single analysis is approaching the level of transcriptomics. However, many problems remain to be solved in protein glycosylation analysis. This mini-review discusses the technical issues of MS2-based glycoprotein identification and efforts to complement them by the Human Glycome Atlas (HGA) Project in Japan.

## Highlights

• This mini-review discusses the technical issues of MS2-based glycoproteomics and efforts to complement them by the human glycome atlas project in Japan.
• Direct identification of glycopeptide by CID MS2 was difficult and less sensitive; however, identification of intact glycopeptide became possible by HCD.
• It is still difficult to identify glycopeptides by MS2-based approaches, since search results are largely different by search engine used.
• To improve low sensitivity of glycopeptide identification, MS1-based extension is effective and chromatographyical pattern helps evaluation of identification by MS2-based search.
• Human glycome atlas project of Japan plans to provide glycoprotein profiles and glycan structures for wide range of human cells, tissues and body fluids as a knowledgebase.

## Introduction

The goals of various avenues of clinical and biological research, such as curing cancer and understanding the function of glycans, remain the same, but the approaches change dramatically in the 30 years since the birth of proteomics [1] as technologies develop. This mini-review discusses current technical problems of MS2-based glycoproteomics and introduces attempts to solve some of them.

## Discussion

(**1**) **Site-specific glycoform (intact glycopeptide) analysis by MS2-based approaches**

**Fig. 1:**
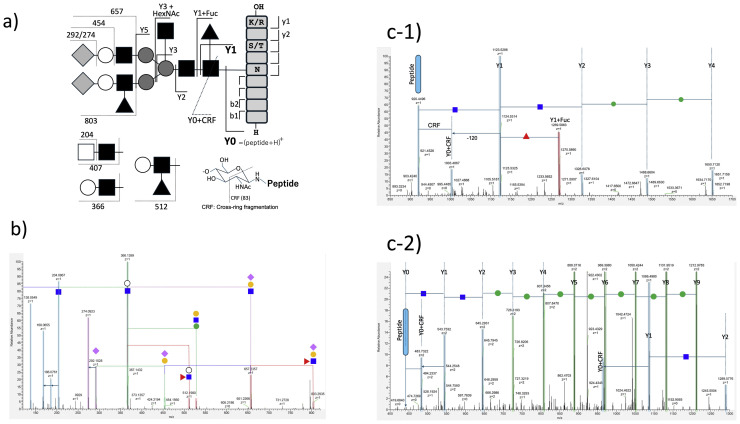
Examples of ions generated by HCD of glycopeptides and representative MS2 spectra. a) Examples of bonds cleaved in glycopeptides and product ions generated. Ions on the non-reducing terminal side generated by glycosidic bond cleavage(s) in the glycan are called B ions, while those on the reducing terminal/peptide side are called Y ions. Ions on the N-terminal side generated by peptide bond cleavage are called b ions, while those on the C-terminal side are called y ions. b) Example of a MS2 spectrum in the low mass region. The B ions shown in a) are observed. Signals at m*/z* 204 derived from HexNAc, m*/z* 366 derived from Hex+HexNAc, m*/z* 292|274 derived from sialic acid, and m*/z* 657 derived from NeuAc-Gal-GlcNAc are commonly observed. The fucose-containing trisaccharide (m*/z* 512) or tetrasaccharide (m*/z* 803) suggests Lewis-type fucose. c1 and c2) Example MS2 spectra in the high mass region. Observation of consecutive ions from the peptide ion (Y0) to Y0+HexNAc (Y1), Y1+HexNAc (Y2), Y2+Hex (Y3), and Y3+Hex (Y4) suggests an N-glycopeptide. If a Y0+CRF signal is observed, Y0 or Y1 can be easily assigned. In the case of high mannose, a step-like signal of Hex may be observed (c-2). Depending on the mass range, it may be observed as monovalent or trivalent. A Fuc-attached Y1 ion suggests the presence of core fucose (c-1), while Y3+HexNAc suggests the presence of bisecting GlcNAc (Fig. 1a).

Glycopeptides are not simply peptides with small modification groups, but conjugates of two oligomers, namely, oligosaccharides and oligopeptides. Since the oligomers have different stabilities, and specifically glycoside bonds are less stable than peptide bonds in glycopeptides, consequently the peptide portion was hardly fragmented by collision-induced dissociation (CID), making identification difficult. Identification was possible by performing a second fragmentation (MS3) via selecting peptide ion (Y0 ion) or ion of peptide with a few saccharides (Y1) generated by the first collision; however, sensitivity was extremely low. Presently, beam type activation (higher-energy collision-induced dissociation (HCD)) made it possible to simultaneously fragment the glycan and peptide portions of glycopeptides, thereby allowing identification of glycopeptide in a single fragmentation. A series of fragment ions generated from N-glycopeptides is illustrated in Fig. 1a, while representative MS2 spectra of glycopeptides are shown in Fig. 1b-c. One or more bonds are dissociated to generate a variety of ions; however, because glycosidic bonds are weaker than peptide bonds, ions generated by glycosidic bond cleavage (B/Y ions) are prominently observed. In the case of N-glycopeptides, ions derived from dissociation at the common trimannosyl core of glycans and from non-reducing terminal glycan motifs extended on the core typically appear (Fig. 1a). Glycan ions derived from fragmentation of the glycan moiety are called B ions (also oxonium ions and diagnostic ions of glycans). Signals at m*/z* 163 of the Hex ion derived from the terminal mannose or galactose, m*/z* 366 derived from LacNAc (Hex+HexNAc), m*/z* 274 and m*/z* 292 derived from sialic acid residues, and m*/z* 138/144/168/186/204 derived from HexNAc are commonly observed. Some fragments are inconclusive but strongly suggest the presence of glycosylation motifs. For example, HexNAc×2 (m*/z* 407) suggests LacdiNAc, while Fuc+Hex+HexNAc (m*/z* 512) suggests a fucose on branch such as Lewis-type or blood-type (H) antigen. When these cleavages occur, ions on the peptide side should be generated at the same time and are called Y ions. The Y0 ion is the ion of the deglycosylated core peptide; however, the Y1 ion, in which the innermost GlcNAc of the chitobiose backbone remains on the peptide, is often observed more strongly. If an N-glycan is attached, the trimannosyl core will be common; therefore, a step-like ion such as peptide+HexNAc(Δm*/z*203)+HexNAc+Hex(Δm*/z* 162)+Hex+Hex…, which suggests an extension from the Y0 ion, will be observed. When three ions are observed at intervals of 203, they are likely Y0–Y2 ions, but this is not always the case. If a product generated by cross-ring fragmentation (CRF) within the innermost GlcNAc ring is seen between the Y0 and Y1 signals (Y0+CRF, Fig. 1a, c-1, and c-2), Y1 (Y0) can be easily assigned. Additionally, Y3 and more extended Y ions are useful to suggest Y0 (peptide mass). An ion suggesting further glycosylation of the trimannosyl core is useful to detect two characteristic glycosylation motifs. The Y1+Fuc fragment suggests the presence of core fucose (Fig. 1a, c-1). Similarly, the Y3+HexNAc fragment suggests the presence of bisecting GlcNAc. The ions remaining after removal of B ions with fixed masses and Y ions with fixed intervals from the complex MS2 signals are likely fragments derived from peptides (b/y ions). By matching these fragments with the predicted precursor mass (e.g., Y0) or performing an open search, the peptide moiety can be identified.

The algorithm of software to search and identify glycopeptides based on MS2 spectra varies depending on the focus and order of the search, but candidates are basically presented assuming the abovementioned fragment structure. However, different identification lists are provided from the same MS data depending on the software used, and many glycopeptides are unique to each software. In a pilot study by the Human Glycoproteomics Initiative (HGI), the successor of the Human Disease Glycomics/Proteome Initiative of the Human Proteome Organization (HUPO), the same mass data were used to compare the results of different software packages, typical discrepancies were identified, and development of better software was promoted while raising awareness among users [2]. In addition to commercially available products including Byonic [3], PEAKS GlycanFinder [4], and Mascot [5], many software packages released by academic researchers are widely available [[6], [7], [8], [9], [10], [11], [12], [13]]. The pGlyco series [[6], [7], [8], [9]], Glyco-Decipher [10], and MSFragger-glyco [11] are some examples, and their performances have been compared [14,15]. MS2-based glycopeptide search algorithms can be categorized into traditional approaches (e.g., Byonic and ProteinProspector) and recently emerged glycan- or peptide-first search types [14,16]. Glyco-Decipher and MSFragger-glyco are categorized as the former and latter, respectively. PEAKS Glycan Search is based on a de novo sequencing algorithm, while GRable (by Glyco-RIDGE) is based on MS1 [17]. I performed simple spot tests using Glyco-Decipher, Byonic, MSFragger-Glyco, and PEAKS-GlycanSearch with common MS data of commercially available serum-derived glycopeptides (which are obtained using Amide-80 column from a tryptic digest of serum after MARS human-7 column depletion). Although this was a limited spot test, 800–1200 types of glycopeptides were identified by each software (data not shown). Glycopeptides identified by two or more software packages accounted for 50 % of the total, and the rest were uniquely identified by one software package. In total, 368 of 1967 glycopeptides were identified by all four software packages. A comparison of these MS2-based methods with the results of MS1-based GRable (approximately 1100 identifications) revealed that 40–50 % of glycopeptides were commonly identified. GRable detects glycoforms with the same core peptide based on glycan heterogeneity, while MS2-based identification often does not identify glycopeptides with the same core. However, such glycoproteins accounted for only 8–16 % of the total and this does not seem to be the main cause of the disparate results. Details will be provided in a follow-up report, but Hogan et al. recommend using a combination of software packages with different approaches, namely glycan-first and peptide-first approaches [18].

(**2**) **Approaches to improve the accuracy of identification**

The identification of glycopeptides is hampered by problems such as low sensitivity and the possibility of misidentification; therefore, efforts are being made to evaluate its reliability from various perspectives and to improve its validity. As pointed out in the abovementioned HGI report and elsewhere, substitutions due to Fuc×2=NeuAc+1 are often seen [2]. The masses of these molecules inherently differ by 1 Da; therefore, this substitution is unlikely to occur if the precursor mass is accurately measured. However, glycopeptides have larger masses than non-glycopeptides. Therefore, the relative intensity of the monoisotopic signal is lower than that of the largest signal, and the signal intensity may decrease to the noise level or even disappear. Based on the assumption that this causes a monoisotopic error, a function automatically corrects the monoisotopic signal mass and attempts to search for a match. It seems appropriate to try to make the monoisotopic mass 1 smaller, but not larger.

There is an effort to evaluate the shape (intensity distribution) of the isotope pattern [17]. There are two ways to do this. One is to evaluate whether the monoisotopic signal is detected correctly, e.g., by checking the degree of agreement with predictions based on the average elemental compositions of peptides estimated from the masses. The other is to evaluate whether the assigned structure matches the signal shape, which is calculated using the specific elemental composition of the determined structure. Isotope signals are not always acquired at a constant intensity ratio; therefore, it is difficult to evaluate the envelope with a single-shot spectrum. Therefore, increasing the number of microscans or using the sum (or average) of multiple spectra is expected to lead to an improvement. Correct monoisotopic mass acquisition is essential for correct identification; therefore, acquisition and correction methods for weak signal monoisotopic masses are expected to be developed.

(**3**) **Approaches to extend MS2-based identification**

**Fig. 2:**
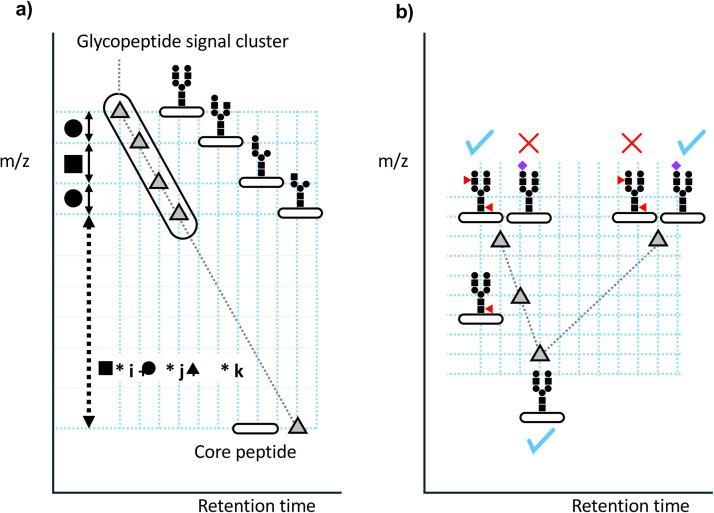
Principle of the Glyco-RIDGE method. a) A group of glycopeptides with a common peptide but different glycans are eluted with similar retention times in reversed-phase LC/MS analysis using a C18 column. Their mass difference corresponds to the mass of the monosaccharides (or disaccharides) that make up the glycan. Based on this feature, glycopeptide signals can be explored and detected as clusters. The core peptide is likely included in the list of peptides identified with high sensitivity by deglycosylation of the same sample. This is because deglycosylation removes heterogeneity and improves the ionization efficiency. The same principle can be applied to the assignment of glycopeptide groups with core peptides and to the clustering between glycopeptides, i.e., the time difference is approximately the sum of each time shift of monosaccharides, and the mass difference is also the sum of the exact masses of monosaccharides. If one cluster member is identified by MS2, the other glycan compositions on the same core can be assigned, even if the other cluster members are not identified by MS2. The process of searching for clusters, matching with the core list, and verifying the certainty of the matching is accomplished by GRable. b) The retention time is slightly reduced by addition of a neutral saccharide and conversely delayed more by addition of sialic acid. If a multiple-fucose assignment is suspected, it may be possible to distinguish it by comparing its apex retention time with that of a glycopeptide of the same core. If the number of sialic acids is the same, the retention times will be similar.

One way to overcome the sensitivity barrier is to use an MS2-independent approach. In the case of glycans, by attaching a restricting moiety to the reducing end, e.g., a fluorescent dye or a peptide derived from a certain protein, the glycan composition can be determined from the whole mass of the glycan or its conjugate. GlycoModTool is an excellent software that estimates the glycan composition of glycans and a few predictable glycopeptides [19]. Peptoonist assigns glycopeptides by combining the masses of glycans in a library with the masses of a limited number of core candidates [20]. To avoid misassignment due to chance, it is necessary to use accurate masses. Nagai-Okatani et al. also tried to develop a method to identify glycopeptides utilizing MS1 in addition to MS2 (Glycan heterogeneity-based Relational Identification of Glycopeptide signals on the Elution profile (Glyco-RIDGE)) and recently published a software to perform the analysis (GRable) [17]. They devised a method that can handle not only one or a few purified (enriched) proteins, but also complex unknown mixtures (Fig. 2a).

Their approach is based on the heterogeneity of glycans and starts with thorough selection of glycopeptide signals that share a common core, using the mass difference of glycopeptides matching the monosaccharide and close retention times in liquid chromatography (for neutral glycans) as indicators. Another software called GlycopeptideGraphMS also utilizes this phenomenon [21]. Glycans are composed of limited components (saccharides), some of which are isobaric, meaning fewer types of glycan components by mass, and human glycans are assumed to be mainly four species: Hex, HexNAc, Fuc, and NeuAc. If the mass difference between the glycopeptide candidates detected based on heterogeneity and the identified core peptide precisely matches the integer multiple sum of these four components, the combination of the core and glycan can be inferred. In practice, there are cases where no signal matches the given core or multiple candidates match. In case of the latter, single candidate must be selected.

Even after selection based on the commonness of matched glycans and the retention time difference of glycopeptides and the core (deglycosylated) peptide, a mistake can still be made in matching; therefore, further confirmation or selection is performed using MS2 information. Step-by-step information is available if MS2 is performed; therefore, it is important that MS2 is performed comprehensively and with high sensitivity for as many weak signals as possible. A match of the peptide mass (Y0 ion or Y series ions that suggest it) is most reliable. In this case, matching of motifs using B ions also supports the match. For example, the possibility of the presence of sialic acid, Lewis fucose, or LDN is increased by the presence of each corresponding B ion. However, the possibility that the precursor ion was not completely isolated should also be considered. Finally, it is necessary to confirm the presence of diagnostic ions (e.g., m*/z* 204), which indicate that the molecules detected by clustering are glycopeptides.

The GRable software performs these validations and provides results ranked according to which criteria they are met. Identification by a method not based on MS2 is unlikely to be acceptable to many researchers; however, because the elution properties of glycopeptides have clear characteristics, it is possible to distinguish whether multiple fucoses or sialic acid are present from the elution positions relative to other components (Fig. 2b). If glycopeptides with the same core are identified, the certainty of the identification can sometimes be evaluated by assessing the relationship between their elution positions and compositions.

(**4**) **Reference map-aided glycopeptide search method**

Advances in analyzers have made it possible to obtain 1 million MS2 spectra in one run. The speed is so fast that a data-independent acquisition (DIA) with a mass range of 4 Da can cover a wide range of precursor ions in a cycle lasting a few seconds. Currently, the Human Proteome Project of HUPO predicts about 20,000 proteins, of which 18,397 (as of October 18, 2024) have been found. When these proteins are digested with trypsin, they are cleaved at an average of 50 sites, yielding 1 million peptides if mis-cleavages and other modifications are not considered. Put simply, if these peptides can be ionized, all present proteins can be identified. In fact, peptides with good ionization efficiencies that represent each protein have been identified, and the number of identifications in proteome analysis has reached 10,000. However, even if MS2-based identification is accelerated and highly sensitive, the identification efficiency of glycopeptides will not be as high as that of proteins because the core is not necessarily a peptide with a good ionization efficiency.

In view of this situation, the Human Glycome Atlas Project (HGA) in Japan, which was launched in 2023, plans to comprehensively identify glycomes of human proteins and provide glycoprotein profiles and glycan structures for each cell or tissue as a knowledgebase. Furthermore, it aims to build a new analysis platform that can easily identify glycopeptides using this structural information. To realize this, it will first perform deep LC/MS analysis of glycopeptides, prepare a list of identified glycopeptides with their retention times and calculated masses (called a reference map), and then build a tool to suggest glycopeptides by comparing researchers' LC/MS data with the map data. A map of commercially available serum and plasma containing >20,000 types of glycopeptides will be generated and a matching software will be built in parallel. Subsequently, maps of various tissues and cells will be generated.

This method is basically the same as the AMRT (accurate mass - retention time)-tag method that was once proposed for proteome analysis [22]. To confirm the feasibility of this method, we first compared the masses of about 15,000 glycopeptides identified from commercially available serum and plasma. Several patterns have exactly the same mass but different structures and therefore cannot be matched to a single candidate. Differences of one oxygen atom are seen between amino acid residues such as Phe-Tyr, Ala-Ser, and Met-Met(oxidized) and between glycans such as Hex-Fuc and NeuAc-NeuGc. Therefore, these combinations cancel out the mass difference, resulting in the possibility of misidentifying a molecule with the same mass but a different structure. For example, there are four subclasses of IgG heavy chains, and the amino acid sequences around the glycosylation site differ as follows: two have the same mass and the other two have a mass difference of one oxygen atom from these. When a glycan is added, even if its structure is different, the overall mass of the glycopeptide may be exactly the same, making it impossible to distinguish them based on mass alone.

IgG1: YnSTY, IgG2: FnSTF, IgG3: YnSTF, and IgG4: FnSTY (n: glycosylated Asn)

**Fig. 3:**
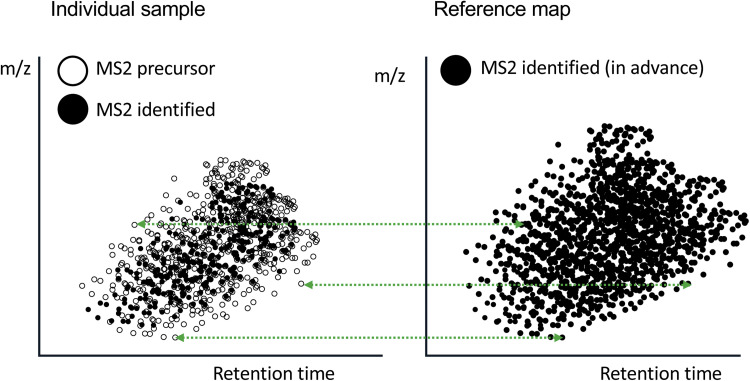
Image of the reference map-aided identification method. When glycopeptides are analyzed by LC/MS, many MS2 spectra are acquired, but only a few are identified. For the remaining MS2 precursors, information on m*/z,* charge, retention time, and some fragment ions is acquired and recorded. In the left panel, identified signals and unidentified precursors are represented by solid and open symbols, respectively. To improve the sensitivity of MS2-dependent analysis, we generated a tool that predicts suitable glycopeptide candidates based on mass and retention time of unidentified precursors. HGA performs comprehensive and high-depth identification in many kinds of human cells, tissues, and body fluids starting with commercially available plasma and serum as samples that are expected to be frequently used in biomarker discovery and other studies to make a series of reference maps. Protein-level fractionation (removal of major proteins), peptide-level fractionation (hydrophilic interaction chromatographic separation), gradient elongation, and repeat analysis will be performed to create reference maps containing information on many of the identified glycopeptides. Users will be able to generate predicted results by providing a set of results from their own analyses (exact mass and retention time of the MS2 precursor and its fragment information, regardless of whether it led to an identification), which will be matched against the data in the reference map (right panel). (EOF).

Additionally, some peptides and glycopeptides have similar, but not identical, masses. Therefore, it is recommended to measure MS1 spectra with high precision (e.g., by using the Lockmass function), but it is difficult to guarantee an error of <2 ppm even with a relatively sophisticated analyzer. Some molecules in the preliminary list (about 15,000 glycopeptides) cannot be distinguished with an error of <2 ppm even if they do not have exactly the same mass. It is possible that molecules that cannot be distinguished by mass information can be distinguished by retention time information; however, these two types of information cannot present a single candidate for some glycopeptides. In this case, a third type of information, namely, MS2 information, is required. There are two main ways to use MS2 information. One is to use the presence of a diagnostic ion (e.g., m*/z* 204) as an indicator that the precursor is a glycopeptide. In a test study, glycopeptides matching the masses of non-glycopeptides were present; therefore, it is important to exclude non-glycopeptides from the search. For this purpose, a filter that discriminates glycopeptides using precise masses (numbers after the decimal point) may be effective [23]. Another is to find information consistent with the structure. The most likely result is that the MS2 search result and the result by mass matching is the same; however, even if identification is not possible, if the peptide mass estimated from Y ions matches the mass of candidate core, then this will be strong support for the selection. From B ions, it is important to match the composition of the candidate with the presence or absence (strength) of component-related fragments, such as those containing fucose or sialic acid. This may also require consideration of isolation interference. Furthermore, searching for y ions will provide a major clue regarding whether the C-terminal residue (y1) is Lys or Arg and what the second residue is. This way of using MS2 information has been incorporated into GRable and shown to be effective [17]. Based on this idea, we are currently establishing a method using AMRT and MS2 information to select and screen candidates by matching them with a reference list (Fig. 3). I believe this method will be able to identify many more signals from a single analysis than can be identified by searching or can at least present candidates, thereby compensating for the low analytical sensitivity. The practicality of reference identification using serum and plasma will be demonstrated elsewhere.

(**5**) **Conclusion and future perspectives**

This mini-review outlines glycoproteomic approaches using MS. The rise of proteomics required not only separation and analysis techniques, but also the development of information technology and protein sequence information. In this context, it is interesting that in the first glycoproteomic paper, glycopeptides obtained by an immobilized lectin column were identified using Edman degradation and the experiment was performed using the nematode Caenorhabditis elegans, the first multicellular organism to have its genome sequenced [24]. This approach was made possible by the facts that nematodes lack sialic acid and peptide glycoforms are eluted in a narrow retention time range by reversed-phase liquid chromatography even though the glycans are different.

MS equipment is evolving at an astonishing speed, and the depth of analysis is increasing daily. However, as mentioned above, glycopeptides are prone to errors in monoisotopic signals due to their large masses. This is also true for normal peptides. However, for glycopeptides, structural features hide this error (or somehow make it match); therefore, it is desirable to develop software that allows identification results to be accepted with confidence or suggests the possibility of misidentification. As the depth (number) of identifications increases, verification cannot be performed manually. Glycans have diverse, heterogeneous, and weak structures, making it difficult to identify glycopeptides. However, this is the inherent nature of glycopeptides; therefore, an approach that utilizes this feature seems interesting, but is useless if it compromises reliability. The development of highly accurate identification methods will continue, but higher-level information than composition, namely, binding sites and binding direction, is required for glycans. Standard products with defined structures are essential for structural analysis. It is hoped that a technology to create these products will be developed in parallel.

## CRediT authorship contribution statement

**Hiroyuki Kaji:** Writing – original draft.

## Declaration of competing interest

The authors declare that they have no known competing financial interests or personal relationships that could have appeared to influence the work reported in this paper.

## Data Availability

No data was used for the research described in the article.
